# Functional Intercellular Transmission of miHTT via Extracellular Vesicles: An In Vitro Proof-of-Mechanism Study

**DOI:** 10.3390/cells11172748

**Published:** 2022-09-03

**Authors:** Roberto D. V. S. Morais, Marina Sogorb-González, Citlali Bar, Nikki C. Timmer, M. Leontien Van der Bent, Morgane Wartel, Astrid Vallès

**Affiliations:** 1 Department of Research and Development, uniQure Biopharma B.V., 1105 BP Amsterdam, The Netherlands; 2 Department of Gastroenterology and Hepatology, Leiden University Medical Center, 2333 ZA Leiden, The Netherlands

**Keywords:** adeno-associated virus, exosomes, extracellular vesicles, gene therapy, Huntington’s disease, intercellular communication, miRNA

## Abstract

Huntington’s disease (HD) is a fatal neurodegenerative disorder caused by GAG expansion in exon 1 of the huntingtin (*HTT*) gene. AAV5-miHTT is an adeno-associated virus serotype 5-based vector expressing an engineered HTT-targeting microRNA (miHTT). Preclinical studies demonstrate the brain-wide spread of AAV5-miHTT following a single intrastriatal injection, which is partly mediated by neuronal transport. miHTT has been previously associated with extracellular vesicles (EVs), but whether EVs mediate the intercellular transmission of miHTT remains unknown. A contactless culture system was used to evaluate the transport of miHTT, either from a donor cell line overexpressing miHTT or AAV5-miHTT transduced neurons. Transfer of miHTT to recipient (HEK-293T, HeLa, and HD patient-derived neurons) cells was observed, which significantly reduced *HTT* mRNA levels. miHTT was present in EV-enriched fractions isolated from culture media. Immunocytochemical and in situ hybridization experiments showed that the signal for miHTT and EV markers co-localized, confirming the transport of miHTT within EVs. In summary, we provide evidence that an engineered miRNA—miHTT—is loaded into EVs, transported across extracellular space, and taken up by neighboring cells, and importantly, that miHTT is active in recipient cells downregulating *HTT* expression. This represents an additional mechanism contributing to the widespread biodistribution of AAV5-miHTT.

## 1. Introduction

Huntington’s disease (HD) is a fatal inherited neurodegenerative disorder driven by a CAG (cytosine, adenine, guanine) trinucleotide expansion in exon 1 of *HTT*, the gene that encodes huntingtin protein. Mutant huntingtin protein (mHTT) contains an abnormally long polyglutamine tract, which confers both the loss of normal function and a toxic gain of function, eventually leading to neuronal cell death [1,2,3,4,5]. HD is characterized by motor, cognitive, behavioral, and psychiatric symptoms, which vary widely amongst patients [1,2,5,6]. The average age at onset is 40 to 50 years, and survival after diagnosis is 15 to 24 years [7,8,9]. The striatum (putamen and caudate) is the initial and primary site of HD pathology [10]. At later stages of the disease, atrophy and cortical thinning progressively spread to diverse regions of the brain [11]. There are no approved disease-modifying treatments for HD [3,5].

Currently, a major focus is the development of drugs targeting huntingtin by reducing *HTT* RNA or protein expression, with the goal of lowering mHTT and mitigating its pathogenic effects [12]. RNA targeting strategies include engineered antisense oligonucleotides (ASOs), small interfering RNAs (siRNAs), expressed short hairpin RNAs (shRNAs), and artificial microRNAs (miRNAs) [12,13]. Preclinical studies in murine HD models show that successful HTT lowering can delay disease progression or reverse the HD phenotype [14,15,16,17]. Next to specificity and potency, a good biodistribution to the most vulnerable regions in HD (the caudate, putamen, and cortical regions) is key for the success of these therapies.

ASO drugs that either selectively downregulate mHTT or lower total HTT are undergoing evaluation in clinical trials. However, these therapies are short-term strategies that rely on frequent intrathecal dosing into the cerebrospinal fluid (CSF), and their biodistribution in key regions involved in HD, such as the caudate and putamen, is unclear [18]. 

In addition to ASOs, the most advanced HTT-lowering strategy is virally delivered miRNA-based gene therapy. AAV5-miHTT is a recombinant, replication-deficient adeno-associated virus (AAV)-based gene therapy comprising a single-stranded DNA genome encoding an engineered miRNA targeting exon 1 of human *HTT* mRNA packaged in an AAV5-derived capsid [17,19]. Two parallel Phase I/II trials of AAV5-miHTT, one in the US [HD-GeneTRX-1 (NCT04120493)] and one in the EU [HD-GeneTRX-2 (NCT05243017)], are ongoing.

AAV vector delivery of engineered miRNAs directly to the brain affords the prospect of a one-time administration offering long-term treatment of HD [20,21]. AAV-delivered miRNA-encoding DNA cassettes are stably maintained extra-chromosomally (as episomes), which explains their long-term persistence and ability to drive durable gene suppression following a single infusion [22,23]. 

Engineered miRNAs utilize existing primary miRNA sequences as scaffolds to express the mature therapeutic miRNA after processing. AAV5-miHTT employs an miR-451 scaffold, which undergoes Dicer-independent processing via Argonaute-2 (Ago2) and further 3′-to-5′ trimming by polyA-specific ribonuclease (PARN) [24,25,26,27,28]. This is different from the majority of miRNAs, in which processing is typically performed by the endoribonucleases Drosha and Dicer [25]. Because of the non-canonical processing, the miR-451-derived mature miRNA is produced without the complementary passenger strand present in canonically produced miRNAs [27], significantly reducing the rate of off-target effects, as previously demonstrated in studies using AAV5-miHTT [29]. Notably, in addition to its non-canonical processing, miR-451 is markedly enriched in extracellular vesicles (EVs) [30].

EVs are a heterogeneous group of membranous nano-scale particles [31], including exosomes, microvesicles, and microparticles, which vary in size, surface marker expression, and biogenic pathways. EVs exhibit surface markers that may reflect the EV subtype and the cell from which they are derived. Tetraspanin family proteins are among the most widely represented membrane proteins of EVs (notably CD9, CD63, and CD81) [32,33,34]. CD9 is regarded as a pan-EV marker [32]. Therefore, these molecules have been adopted as characteristic surface markers [33,34]. 

EVs can transport complex cargos, including nucleic acids, proteins, and lipids [35]. Analysis of circulating RNAs demonstrated that miRNAs are the most abundant EV-associated RNA species [36]. The EV miRNA content may differ from that of the “parent” cell, implying that miRNA loading is an active and selective process, dependent on cellular sorting mechanisms to regulate packaging into EVs [37,38].

Preclinical studies conducted in a variety of HD models, including rodents, minipigs, and non-human primates (NHPs), indicate that intrastriatal injection of AAV5-miHTT mediates safe and sustained vector DNA and miRNA expression, leading to *HTT* mRNA and protein suppression. Functional improvements and increased survival were also observed [17,20,39,40,41].

AAV5-miHTT biodistribution studies consistently revealed transduction of brain regions beyond the target surgical areas (putamen and caudate nucleus) [20,39,41]. The extensive distribution of AAV5-miHTT is key for therapeutic efficacy, given the widespread pathology in HD [11]. AAV5 serotype vectors can be disseminated by both retrograde and anterograde axonal transport from their original depot to other brain regions [42,43,44]. The propensity for AAV transport within the nervous system is, in part, serotype dependent [45], with AAV5 demonstrating more efficient axonal transport than other serotypes such as AAV2 [45,46]. How efficiently AAV vector transport occurs through neuronal connections in the context of HD-related neurodegeneration, or whether other mechanisms contribute to its biodistribution beyond target regions, remains unknown. Previously, we have shown that AAV5-delivered miRNA molecules were released from HD patient-derived neuronal cells in association with EVs [47]. In addition, EV-associated miHTT was documented in CSF of NHPs two years after a single intrastriatal infusion of AAV5-miHTT [47].

Our working hypothesis is that miHTT can be loaded into EVs, supporting the widespread biodistribution of AAV5-miHTT. Here, we performed in vitro studies in different cell models to assess the EV loading, cell-to-cell, and functional transfer of miHTT. Our results show that miHTT is enriched in EVs and support the concept that EV-mediated miHTT transport is an additional mechanism, alongside AAV5-miHTT axonal transport, contributing to the widespread biodistribution of miHTT. 

## 2. Materials and Methods

### 2.1. miHTT-Overexpressing Stable Cell Line Generation

The HEK293-miHTT stable cell line was generated using the Flp-In™ System (Thermo Fisher Scientific, Waltham, MA, USA). After gene synthesis (GeneArt^®^ (Thermo Fisher Scientific, Waltham, MA, USA)), the miHTT sequence (Supplementary Materials and Methods: Sequence information) was subcloned into a modified pcDNA™5/FRT expression plasmid (Thermo Fisher Scientific, Waltham, MA, USA). Flp-In^TM^293 cells were transfected with DNA plasmids containing the miHTT construct under the control of the cytomegalovirus (CMV) promoter (Thermo Fisher Scientific, Waltham, MA, USA). Drug-resistant cells were expanded following the addition of selection agents. HEK293-miHTT stable pool generation was confirmed by RT-PCR using TaqMan prime/probe (assay ID #CTXGPY4 (Thermo Fisher Scientific, Waltham, MA, USA)). No miHTT was detected in wild-type HEK-293T cells (Supplementary Table S1). A U6 probe (assay ID #001973 (Thermo Fisher Scientific, Waltham, MA, USA)) was used as a positive control (Supplementary Table S1). 

### 2.2. Differentiation of Forebrain Neuronal Cultures from Human HD-Induced Pluripotent Stem Cells (iPSCs)

Human iPS cells (ND42229*B) containing 71 CAG repeats were reprogrammed from HD patient-derived fibroblasts (Coriell Institute Stem Biobank, Camden, NJ, USA) [47]. These cells were differentiated and maintained in culture as previously described [47].

### 2.3. AAV5-miHTT Vector Production

AAV5-miHTT is an AAV serotype 5 vector that contains an miHTT expression cassette under the control of a combination of the cytomegalovirus early enhancer element and chicken β-actin promoter (CAG promoter). The vector was manufactured in insect cells using a baculovirus expression system as previously described [24]. Post-infection, the cell lysate was purified on the AKTA explorer FPLC chromatography system (GE Healthcare, Chicago, IL, USA) using AVB sepharose (GE Healthcare, Chicago, IL, USA) [40]. After purification, the concentration of AAV vector genome copies (gc) (gc/mL) was determined by qPCR amplification using a primer pair binding to the CAG promoter [24]. Additional QC testing included sub-visible particle testing and determination of possible contaminants, e.g., endotoxins and bioburden.

### 2.4. Transduction of HD Patient iPSC-Derived Neurons with AA5-miHTT 

HD patient iPSC-derived neurons were transduced with different doses of AA5-miHTT: 3 × 10^11^ (genome copies (gc)), 3 × 10^12^ gc, and 3 × 10^13^ gc, corresponding to multiplicities of infection (MOIs) of 1 × 10^5^, 1 × 10^6^, and 1 × 10^7^, respectively. The medium from neuronal cultures was refreshed every two days. On day 5 and day 12 after transduction, the conditioned medium was collected and centrifuged at 3000× *g* for 15 min to remove cells and cell debris. 

### 2.5. EV Isolation from Culture Medium 

#### 2.5.1. EV Isolation by Precipitation

EVs produced and released into the culture medium by AA5-miHTT transduced HD patient iPSC-derived neurons (as per Section 2.4) were isolated with ExoQuick-TC (System Bioscience, Alto, CA, USA) according to the manufacturer’s protocol. In total, 3 mL of ExoQuick buffer was added to 10 mL of conditioned medium and incubated at 4 °C overnight. 

The following day, EVs were collected by centrifugation at 1500× *g* for 30 min, and the supernatant was discarded. The residual solution was additionally centrifuged at 1500× *g* for 10 min, and the EV pellets were subsequently re-suspended. These EV preparations were used to treat naïve HD patient iPSC-derived neurons (Section 2.6).

#### 2.5.2. EV Isolation Using Size-Exclusion Chromatography (SEC) 

Isolation of EVs from HEK293-miHTT cell line conditioned culture media was performed as previously described [47]. Briefly, culture medium was collected and centrifuged at 4000× *g* for 15 min at room temperature to remove cells and cell debris. Separation of EVs from protein complexes was achieved by SEC with qEV10 columns (Izon Science, Christchurch, New Zealand). Columns were washed and equilibrated with sterilized 1× D-PBS. Medium (10 mL) was subsequently loaded onto the column, and 26 fractions of 0.5 mL were collected. The EV-enriched media fractions 6, 7, and 8 [47] were concentrated to 300 µL by AmiconVR Ultra-15 Centrifugal Filter Units with a 10 kDa molecular weight cut-off (Merck Millipore, Burlington, MA, USA) by centrifugation at 4000× *g* for 15 min at 4 °C. The entire procedure was performed on conditioned culture media from 3 different flasks. For miRNA detection, 300 µL TRIzol (Thermo Fisher Scientific, Waltham, MA, USA) was added to each fraction, and RNA was isolated using Direct-zol^TM^ (Zymo Research, Irvine, CA, USA) according to the manufacturer’s protocol. miHTT levels were analyzed by RT-qPCR and small RNA sequencing (Section 2.9). 

### 2.6. Functional miRNA Transfe—via EVs—to Naïve HD Patient iPSC-Derived Neurons

EVs (prepared by precipitation as previously described in Section 2.5) from AA5-miHTT transduced HD patient iPSC-derived neurons were pooled, serially diluted (0.1×, 0.5× 1×, 2×, or 5×), and 1 ml of these dilutions was added to 1 × 10^5^ naïve HD patient iPSC-derived neurons per well of a 24-well plate. EVs precipitated from PBS-treated HD patient iPSC-derived neurons were prepared and diluted as described above and used as controls. Cells were harvested 24 h after EV transfer for miHTT detection by RT-qPCR. 

### 2.7. In Vitro Contactless Co-Culture Transwell Assay 

#### 2.7.1. HD Patient iPSC-Derived Neurons 

A contactless co-culture transwell assay was developed to investigate miRNA transfer through EVs and subsequent mRNA target engagement. HD patient iPSC-derived neurons (regarded as donor cells) transduced with AAV5-miHTT (3 × 10^13^ gc), as previously described, were seeded into transwell polyester membrane cell culture inserts (24 mm, 0.4 μm pore) (Sigma-Aldrich, St Louis, MO, USA).

Naïve HD patient (regarded as recipient cells) iPSC-derived neurons (5 × 10^5^ cells per well) were seeded in a 6-well plate. After 48 h, transwell inserts were placed on top of the wells, and cells were co-cultured for 2 weeks (n = 6). The medium was refreshed every 2 or 3 days. Recipient cells cultured without inserts were used as controls. Cells were harvested with Accutase (Sigma-Aldrich, St Louis, MO, USA) for molecular analysis, including AAV5 genome copy determination and *HTT* mRNA analysis by RT-qPCR.

#### 2.7.2. HEK-293T and HeLa Cells 

HEK-293T (Sigma-Aldrich, St Louis, MO, USA), HeLa (ATCC, Manassas, VA, USA), and HEK293-miHTT (generated as previously described) cell lines were maintained in Dulbecco’s Modified Eagle’s Medium (DMEM) (Thermo Fisher Scientific, Waltham, MA, USA) supplemented with 10% fetal calf serum, 100U/mL penicillin and 100U/mL streptomycin (Gibco^TM^ (Thermo Fisher Scientific, Waltham, MA, USA)), at 37 °C and 5% CO_2_. 

The co-cultured cells were prepared as described: HEK-293T or HeLa cells, regarded as recipient cells, were seeded into the bottom of a 6-well plate and allowed to attach. HEK293-miHTT cells, regarded as donor cells, were placed on the bottom of a transwell polyester membrane cell culture insert (24 mm, 0.4 μm pore) (Sigma-Aldrich, St Louis, MO, USA) using complete DMEM. After cell seeding, transwell inserts were placed into the 6-well plates containing the recipient cells. All cultures were maintained at 37 °C in a humidified 5% CO_2_ incubator for 4, 6, or 9 days in complete DMEM medium. For the longest (9-day) period of co-culture, cells were incubated with complete DMEM medium containing a cell cycle inhibitor (10 nM sodium butyrate (Sigma-Aldrich, St Louis, MO, USA)). As controls, cells were seeded separately on the bottom of a 6-well plate, or donor cells were placed into the inserts without recipient cells in the bottom of the 6-well plate. Each timepoint had a minimum of 6 repeats per experiment. At the end of the experiments, samples were processed for RNA isolation. 

### 2.8. Vector DNA, miHTT, and HTT mRNA Measurement by RT-qPCR

For viral vector DNA isolation, neuronal cells were processed using a DNeasy Blood & Tissue Kit (69504 (Qiagen, Valencia, CA, USA)) following the manufacturer’s protocol. AAV5 vector genome copies were measured by qPCR using a SYBR Green protocol (Applied Biosystems, Foster City, CA, USA) and a validated standard line for detection of the CAG promoter. The forward and reverse primer sequences used were GAGCCGCAGCCATTGC and CACAGATTTGGGACAAAGGAAGT, respectively. The standard line was used to calculate the genome copies per microgram of DNA. 

Total RNA was isolated from samples using Direct-zol^TM^ (ZY-R205 (Zymo Research, Irvine, CA, USA)) according to the manufacturer’s protocol. cDNA was synthesized using the TaqMan^TM^ MicroRNA Reverse Transcription Kit (4366596 (Thermo Fisher Scientific, Waltham, MA, USA)). To determine miHTT expression levels, a two-step RT-qPCR was performed using TaqMan Fast Universal PCR Master Mix with a customized miHTT primer/probe (assay ID #CTXGPY4 (Thermo Fisher Scientific, Waltham, MA, USA)). The expression levels of miHTT were calculated based on a standard line prepared with synthetic RNA oligos (Integrated DNA Technologies, Coralville, IA, USA).

To measure mRNA lowering and to determine the expression levels of housekeeping genes, cDNA was generated using the Maxima Synthesis kit (K1672 (Thermo Fisher Scientific, Waltham, MA, USA)). cDNA was analyzed by RT-qPCR using TaqMan probes: human *HTT* (assay ID # Hs00918178_m1), human UBC (assay ID Hs00824723_m1), human GAPDH (assay ID #Hs02758991_g1), human ACTB (assay ID #Hs01060665_g1), and human GUSB (assay ID # Hs00939627_m1) (Thermo Fisher Scientific, Waltham, MA, USA). RNA expression was calculated using the ΔΔCT method normalized to a housekeeping gene or to the geometric mean of the housekeeping genes [48].

### 2.9. Small RNA Sequencing 

Total RNA from HEK293-miHTT (donor), HEK-293T, HeLa (control and recipient) cells, and donor cell HEK293-miHTT EV-enriched cell-conditioned medium fractions underwent small RNA sequencing (GenomeScan, Leiden, The Netherlands). Each experimental condition was analyzed in duplicate or triplicate. 

RNA quality and integrity were determined using the Bioanalyzer 2100 (Agilent, Santa Clara, CA, USA) with the RNA 6000 Nano kit (Agilent, Santa Clara, CA, USA) using the 2100 expert vB.02.10.SI764 software (Agilent, Santa Clara, CA, USA). Library preparation was performed using the NEBNext multiplex small RNA library kit for Illumina (Index Primers 1-48, #7560S) (New England Biolabs, Ipswich, MA, USA). Possible adapter dimers were removed using the Blue Pippin size selection system (Sage Science, Beverly, MA, USA). Paired-end 150 bp sequencing was performed using the Novaseq6000 (Illumina Inc., San Diego, CA, USA) according to the manufacturer’s instructions, generating at least 20 million reads per sample. Image analysis, base calling, and quality checks were performed using the Illumina data analysis pipeline RTA3.4.4 and Bcl2fastq v2.20 (Illumina Inc. San Diego, CA, USA). 

### 2.10. Fluorescent In Situ Hybridization (FISH) and Immunocytochemistry (ICC) 

miHTT-overexpressing cells (HEK293-miHTT) were seeded into 3-well chamber slides (iBidi, Gräfelfing, Germany) coated with poly-D-lysin (1:40, Merck, Darmstadt, Germany) and laminin (1:100, Merck, Darmstadt, GE) at a density of 1 × 10^5^ cells per well, 48 h prior to fixation in 10% formalin. After fixation, cells were washed in Dulbecco’s phosphate-buffered saline (D-PBS) (Thermo Fisher Scientific, Waltham, MA, USA) and dehydrated in serially diluted ethanol, and stored at −20 °C in absolute ethanol prior to FISH and/or ICC. 

For EV visualization by ICC, selected antibodies (Table 1) were tested separately or in combination (as antibody cocktails) to optimize the staining protocol. Cells were permeabilized in 1x D-PBS 0.1% Tween 20 (Sigma-Aldrich, St Louis, MO, USA), and non-specific binding was blocked by incubation in 1x D-PBS with 5% BSA (Millipore, Burlington, MA, USA) and 5% Normal Donkey Serum (NDS) (Abcam, Cambridge, UK) for 1 h at room temperature. Cells were incubated with primary antibodies diluted in 1x D-PBS containing 1% BSA and 1% NDS in a humidified chamber overnight at 4 °C, washed, and incubated with the secondary antibodies (Table 1) for 1 h at room temperature. Cell nuclei were counterstained with DAPI (Advanced Cell Technologies, Newark, CA, USA) and mounted with ProLong Gold Antifade medium (Thermo Fisher, Waltham, MA, USA). Negative controls were generated by omitting the primary antibodies. 

To detect miHTT within HEK293-miHTT cells, FISH staining was performed using a miRNAscope kit (Advance cell technologies Inc, Newark, CA, USA) used as per the manufacturer’s instructions. 

In brief, endogenous peroxidase activity was blocked with hydrogen peroxide, and cells were permeabilized with diluted Protease III. FISH staining was performed using two probes specifically designed to target miHTT: one targeting the 30 nucleotide isoform (SR-miHTT30-S1, ref. 1063171-S1) and another the 24 nucleotide miHTT isoform (SR-miHTT24-S1, ref. 1063161-S1) (Supplementary Materials and Methods: Sequence information). As negative and positive controls, respectively, the SR-scrambled-S1 (ref. 727881-S1) and the SR-RNU6-S1 (ref. 727871-S1) probes were used. miRNAs were detected using the Fast Red Reagent kit.

For colocalization of EV-marker signals and miHTT, the FISH staining protocol was followed by ICC using a cocktail of primary antibodies (CD9, CD63, and CD81). After nuclear counterstaining with DAPI, samples were mounted with ProLong Gold Antifade medium (Thermo Fisher, Waltham, MA, USA) and stored at 4 °C prior to image analysis.

### 2.11. Imaging Acquisition and Quantification Analysis 

Images were acquired using a Cell Observer (Zeiss, Jena, Germany) equipped with a Colibri7 light source (Zeiss, Jena, Germany). A 385 nm LED (32.27% intensity, 30 ms exposure time, and a depth of focus of 0.72 µm) was used for the nuclear counterstain. A 568 nm LED (48.87% intensity, 300 ms exposure time, and a depth of focus of 0.93 µm) was used for the FISH analysis of miRNA. A 630 nm LED (52.13% intensity, 250 ms exposure time, and a depth of focus of 1.03 µm) was used for EV-markers. For spectral separation, a 90HE Multiband filter cube (Zeiss, Jena, Germany) was used with a beamsplitter at 405 and 493 nm and Emission QBP 425/30, 514/30, 592/25, 709/100, allowing the separation of all three channels. All images were acquired using a Plan-Apochromat 63x/1.40 NA oil M27 objective (FWD + 0.19 mm) with Immersol 518F (Zeiss, Jena, Germany). The detection system was a 16 bit ORCA-Flash4.0 V3 sCMOS-camera (Hamamatsu Photonics, Shizuoka, Japan) with a pixel array of 2048 × 2048 and 6.5 × 6.5 pixel/µm^2^ with a 1 × 1 binning. Images were saved uncompressed. To determine colocalization of the miRNA and EV-markers, the ApoTome 2 (DOI: 10.1117/1.3083439/Zeiss, Jena, Germany) was used. Optical sectioning was performed with the high grid (428.99L/mm) and 5 phases to acquire 15–18 focal planes with a z-stack distance of 0.255 µm for each channel. The acquired images were deconvoluted in a post-process using the Zen Pro v3.6 software (Zeiss, Jena, Germany), and the single z-planes were exported to HALO v3.4 (Indica Labs, Albuquerque, NM, USA). For image analysis using area quantification, the FL plugin v2.1.7 was used to quantify the area of miRNA, EV-marker signals, and double positive pixels in µm^2^ for each focal plane/image. For analysis, an image-zoom of 1 (full-resolution, 16 bit, 2048 × 2048 px with xyz-resolution of 0.103 × 0.103 × 0.240 µm per pixel) was selected with a minimum intensity threshold of 2580RFU for miRNA and 2899RFU for EV-markers. The average total area (μm^2^), obtained from the entire z-stack of double-positive pixels, was normalized to the total area of miHTT-positive pixels and plotted in Prism v.9 (GraphPad Software, San Diego, CA, USA).

### 2.12. Statistical Analysis

All data are represented as mean ± SEM. Significant differences between 2 groups (i.e., controls versus recipients) were identified using an unpaired Student’s *t*-test. One-way ANOVA multi-test comparison was used to compare the percentage of miHTT isomiRs in samples from the contactless co-culture study in HEK293-miHTT donor cells, HEK-293T, and HeLa recipient cells. GraphPad Prism 8.0 (GraphPad Software Inc, San Diego, CA, USA) was used for all statistical analyses. A value of *p* < 0.05 was considered to be significant.

## 3. Results

### 3.1. AAV-Produced miHTT Is Secreted within EVs and Transferred between Neuronal Cells 

**Figure 1:**
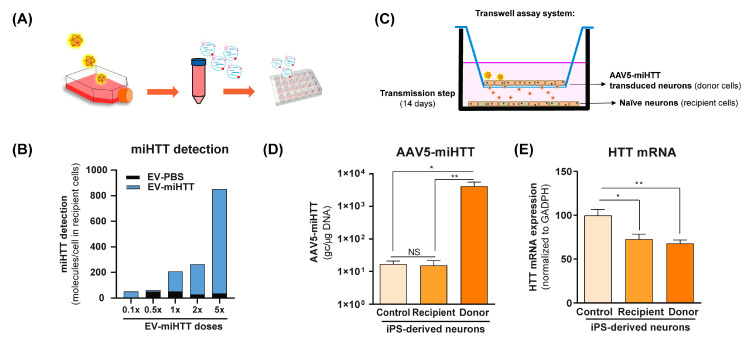
AAV5-miHTT transduction results in EV-associated secretion of miHTT molecules and *HTT* mRNA lowering in naïve HD patient iPSC-derived neurons. (**A**) Schematic depicting the transduction of HD patient-derived iPSC neurons with AAV5-miHTT. EVs secreted from these cells were isolated at days 5 and 12 post-transduction and were subsequently added to naïve HD patient iPSC-derived neurons. (**B**) Pooled and serially diluted EVs (obtained from transduced HD patient iPSC-derived neurons) were added to naïve HD patient iPSC-derived neurons, and miHTT detection was performed by RT-qPCR. N = 1 replicates per experimental condition. (**C**) Schematic depicting a contactless transwell assay using naïve HD patient iPSC-derived neurons as recipient cells and AAV5-miHTT transduced HD patient iPSC-derived neurons. Negative control preparations were naïve HD patient iPSC-derived neurons. Cells were cultured for 2 weeks and subsequently assessed for (**D**) AAV5-miHTT genome copy number. N = 6 replicates per experimental condition, and (**E**) *HTT* mRNA knockdown using RT-qPCR. N = 6 replicates per experimental condition. (* *p* < 0.02, ** *p* < 0.002, NS: non significance).

To investigate the functional transfer of miHTT to naïve cells, a contactless co-culture transwell system was developed. HD patient iPSC-derived neurons that had previously been transduced with AAV5-miHTT were maintained in culture, and the EVs derived from these cells (EV-miHTTs) were isolated and subsequently added to naïve HD patient iPSC-derived neurons (Figure 1A). EV-miHTTs were added (0.1× to 5× dose), resulting in dose-dependent expression of miHTT in the recipient HD patient iPSC-derived naïve neuronal cultures (Figure 1B). Contactless transwell assay experiments were conducted using naïve HD patient iPSC-derived neurons as recipient cells and AAV5-miHTT transduced HD patient iPSC-derived neurons as donor cells (Figure 1C). Cells were maintained in culture for 2 weeks and subsequently assessed for their AAV5-miHTT genome copy number and *HTT* mRNA levels. No viral vector genome transfer between donor and recipient cells was observed, as only donor cells in transwell inserts contained high levels of AAV5 genome copies detected by qPCR (Figure 1D). Significant *HTT* mRNA lowering was observed in recipient HD patient iPSC-derived neurons, confirming the transmission of functional miHTT between cells. Interestingly, in both donor transduced cells and recipient naïve cells, a 30% and 20% *HTT* mRNA lowering was observed, respectively, when compared with control cells (Figure 1C–E).

### 3.2. Stable Cell Line Successfully Overexpresses miHTT 

**Figure 2:**
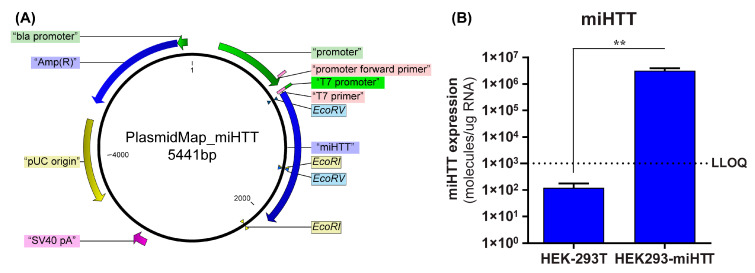
Generation of HEK293-miHTT—a stable cell line overexpressing miHTT. (**A**) Plasmid map of the miHTT sequence cloned into pcDNA™5/FRT plasmid. (**B**) miHTT detection in wild-type HEK-293T (negative control) and in the HEK293miHTT stable cell line. ≥N = 6 repeats per experimental condition. LLOQ, lower limit of quantification. (** *p* < 0.01).

To confirm that the loading and transfer of miHTT via EVs also occurs in an AAV-independent manner, a custom-made cell model was used. A stable cell line (HEK293-miHTT) was, therefore, generated that overexpressed the exogenous miRNA, miHTT, under the control of a CMV promoter (Figure 2A). RT-qPCR analysis demonstrated that miHTT was expressed at a high level by the HEK293-miHTT cells while in unmodified HEK-293T, miHTT expression was below the lower limit of quantification (LLOQ) of the assay (Figure 2B). These data support the use of this cell model for assessing the loading and transfer of miHTT via EVs. 

### 3.3. miHTT Secreted by Donor Cells Effectively Mediates Endogenous HTT-mRNA Lowering in Recipient Cells 

**Figure 3:**
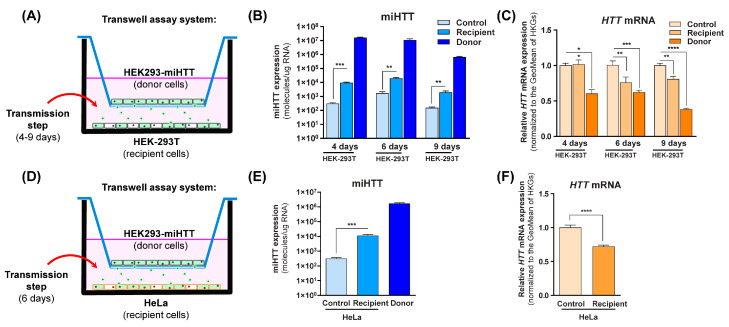
Engineered miHTT is transferred from donor to recipient cells and lowers *HTT* mRNA levels. (**A**,**D**) Schematic description of contactless transwell assay employing both HEK-293T and HeLa cells. (**B**) Contactless transwell assay temporal study of miHTT transfer from the miHTT-over expressing donor cell line to recipient HEK-293T cells at 4, 6, and 9 days of co-culture; ≥N = 6 repeats per experimental condition, and (**C**) the resultant lowering of *HTT* mRNA; ≥ N = 6 repeats per experimental condition. (**E**) Contactless transwell assay study of miHTT transfer from the miHTT-overexpressing donor cell line to recipient HeLa cells at 6 days; ≥N = 6 repeats per experimental condition and (**F**) the resultant lowering of *HTT* mRNA at 6 days; ≥N = 6 repeats per experimental condition. miHTT detection and *HTT* mRNA expression were assessed using RT-qPCR. Geo, geometric; HKGs, housekeeping genes. (* *p* < 0.02, ** *p* < 0.01, *** *p* < 0.001, **** *p* < 0.0001).

Co-cultures were prepared as shown in Figure 3A,D, where HEK293-miHTT were used as donor cells, and HEK-293T or HeLa were used as naïve recipient cells. To determine when miHTT transfer occurs, a time-course experiment was performed using HEK-293T cells as recipient cells (Figure 3B). 

In comparison to their respective controls, significantly increased miHTT levels (determined by RT-qPCR) were observed in the recipient cells at all experimental time points (Figure 3B), indicating that miHTT is taken up by recipient cells. To exclude the possibility of cell migration through the transwell system inserts, an experiment was conducted where media alone was placed at the bottom of the 6-well plate. The samples from those wells were submitted to the same treatment as the ones containing cells. After RNA isolation, no nucleic acid (ng/µL) was measured by NanoDrop spectrophotometry (Supplementary Figure S1), suggesting that there was no cell migration between compartments. 

To determine the effect of miHTT uptake, *HTT* mRNA levels were measured using RT-qPCR in all cells under various experimental conditions. As expected, HEK293-miHTT (donor cells) showed lower *HTT* mRNA levels with respect to control HEK-293T cells at all time points (Figure 3C). At days 6 and 9, a significant reduction was seen in recipient HEK-293T cell *HTT* mRNA levels when compared with controls (*p* < 0.01), suggesting that there is an optimal time frame for in vitro miHTT transfer and mRNA target engagement (Figure 3C). The secretion and uptake of miHTT was, therefore, examined in all subsequent experiments at 6 days of co-culture. In recipient HeLa cells, significantly increased miHTT (*p* < 0.001) was also observed at 6 days when compared with control cells (Figure 3E). As observed in HEK-293T recipient cells (Figure 3C), those levels were sufficient to induce a significant (*p* < 0.0001) reduction (Figure 3F) in endogenous *HTT* mRNA levels in recipient HeLa cells when compared with controls.

### 3.4. FISH and ICC Analyses Provide Evidence for miHTT Transport by Extracellular Vesicles

Contactless co-culture experiments demonstrate the passage of miHTT from donor to recipient cells and the subsequent functional repression of HTT. To explore whether miHTT is trafficked via EVs, fluorescent in situ hybridization (FISH) and immunocytochemistry (ICC) assays to localize EV markers and miHTT sequences were developed. 

EVs have a high content of tetraspanin family proteins, notably CD9, CD63, and CD81; these molecules are widely used as characteristic EV markers [32,33,34]. Supplementary Figures S2 and S3, respectively, show optimized single anti-CD9, CD63, CD81, and triple antibody “cocktail” staining, and FISH localization of miHTT sequences within HEK293-miHTT cells. Negative and positive controls for both ICC and FISH staining are shown in Supplementary Figure S4. 

**Figure 4:**
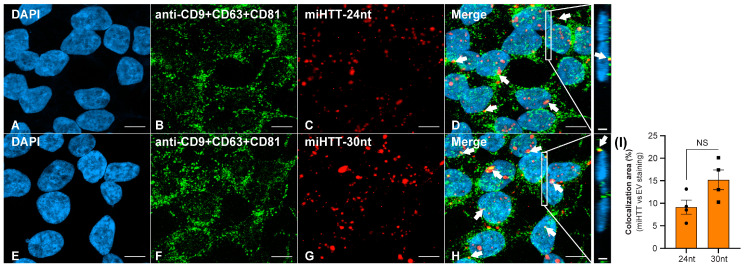
Fluorescent in situ hybridization (FISH) and immunocytochemical (ICC) staining in the HEK293-miHTT cell line for visualization and colocalization analysis between miHTT and EV-marker signals. (**A**–**H**) Visualization of EV-marker signals (green) using a cocktail of antibodies (anti-CD9 + CD63 + CD81). Detection of miHTT (red), using miHTT-24nt or miHTT-30nt probes. White arrows indicate areas of colocalization (yellow) between EV-marker signals and miHTT miRNAs. (**I**) Quantification of EVs and miHTT colocalization area (µm^2^). (**D**,**H**) Inserts show orthogonal projections (xz view). For each single focal plane, the total area (μm^2^) of red (miHTT), green (EVs), and yellow (overlap red: green) pixels were quantified. The total area (μm^2^) of double-positive pixels was normalized to the total area of red pixels. Scale bars main panels A–H = 10 µm. Scale bars insert panels within D & H = 2 µm. nt, nucleotide; NS, non-significant.

FISH staining demonstrated that both the 30 nucleotide and 24 nucleotide miRNA isoforms (Figure 4C,G) were successfully detected in the HEK293-miHTT cell line. ICC staining with a triple cocktail of anti-EV markers demonstrated EVs (Figure 4B,F) within HEK293-miHTT cells. Colocalization (seen in yellow, Figure 4D,H) of the miHTT signal with EV markers implies the carriage of both these miRNA isoforms by EVs. The proportion of total miHTT co-localized with EV markers was predominantly the 30 nucleotide (15%) isoform versus the 24 nucleotide miHTT (9%) isoform (Figure 4I). FISH and ICC analysis provide further evidence of miHTT transfer by extracellular vesicles. 

### 3.5. Small RNA Sequence Analysis Reveals Different miHTT isomiR Profiles in Donor and Recipient Cells and in EV-Enriched Medium Fractions 

**Figure 5:**
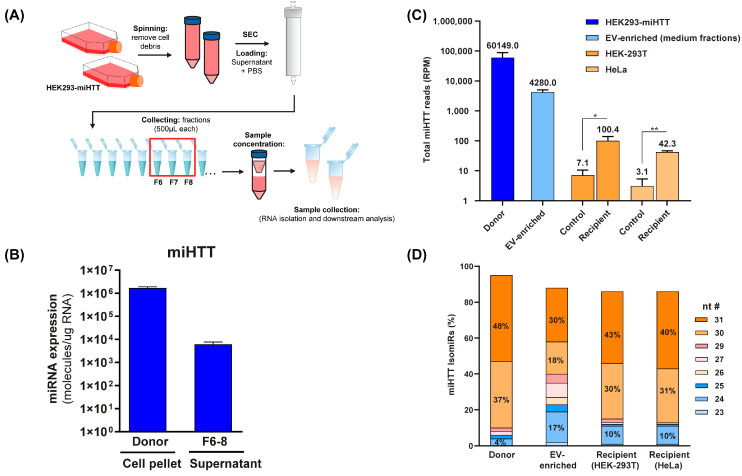
Small RNA sequence analysis reveals different miHTT isomiR profiles in donor cells, recipient cells, and EV-enriched medium fractions. (**A**) Schematic depicting the EV isolation and enrichment process. (**B**) miHTT detection (by RT-qPCR) in donor cell pellets and in EV-enriched cell culture medium fractions (F6-8) isolated by size exclusion chromatography. ≥N = 6 repeats per experimental condition. (**C**) Total RNA sequencing reads were performed on samples from the contactless co-culture study; HEK293-miHTT donor cells, HEK-293T and HeLa recipient cells, and EV-enriched supernatant. N = 3 repeats per experimental condition. (**D**) Percentage of miHTT isomiRs (assessed using small RNA sequencing) in samples from the contactless co-culture study; HEK293-miHTT donor cells, HEK-293T, and HeLa recipient cells. N = 3 repeats per experimental condition. nt #, nucleotide number; RPM, reads per million.

To determine the level of miHTT maturation/processing in the co-culture transwell system, and to ascertain which were the most abundant miHTT isoforms in donor and recipient cells, small RNA sequencing analysis and mature miHTT-specific RT-qPCR were performed (Figure 5A). To this end, EV fractions were obtained from HEK293-miHTT cell culture medium following size exclusion chromatography. RT-qPCR analysis and small RNA-sequencing (Figure 5C) confirmed that miHTT therapeutic molecules were present in the EV-enriched fractions (Figure 5B). 

Small RNA sequencing analysis revealed different miHTT isomiR expression profiles in donor and recipient cells and EV-enriched culture medium fractions (Figure 5D). Our previous studies also showed that pre-miHTT Dicer-independent processing results in mature miHTT of different lengths [29]. Donor cells contained a higher percentage of pre-miHTT, whereas recipient cells contained miHTT that was further processed (Figure 5D); however, these differences were not statistically significant. In EVs (isolated from culture medium), both pre-miHTT and mature miHTT were detected (Figure 5C,D), which confirms the previous observations made in the FISH and ICC studies.

## 4. Discussion

Currently, AAV5-miHTT is being evaluated in the first clinical studies (HD-GeneTRX-1 (NCT04120493); HD-GeneTRX-2 (NCT05243017)) for gene therapy in HD patients. AAV5-miHTT is delivered by a single intrastriatal injection, successfully targeting the most affected brain areas (i.e., striatum and cortex) [20]. As HD progresses, neuropathology extends to other areas [11], necessitating widespread delivery to ensure therapeutic efficacy. Biodistribution studies consistently demonstrate that AAV5-miHTT transduces brain regions beyond the targeted areas mediating *HTT* mRNA and protein suppression [20,39,41], in line with the retrograde and anterograde transport of AAV5 [44]. Whether the axonal-transport-mediated biodistribution will be equally effective in an HD brain undergoing neurodegeneration is still under discussion. In HD minipigs, HTT protein lowering was also observed in areas with low concentrations of AAV5-miHTT vector DNA [20], implying additional mechanisms of spread are operating alongside AAV vector-based transport/transduction. 

Few studies have been conducted in other large animal models using AAV-delivered engineered miRNAs targeting HTT. An AAV9-delivered miRNA targeting human HTT based on an endogenous miR-155 scaffold delivered to transgenic HD sheep demonstrated a local transduction pattern [49]. McBride et al. engineered an AAV2/1 vector to deliver an HTT suppressing miRNA cloned into an artificial human miR-30 scaffold. Following striatal injection, localized HTT suppression was documented in the NHP striatum. However, this study was largely intended to document the safety and tolerability of HTT suppression in NHPs rather than biodistribution [50]. In general, these studies using different capsids and miRNA scaffolds to deliver the therapeutic HTT targeting miRNA show a less widespread biodistribution than AAV5-miHTT.

We hypothesize that multi-modal dissemination of AAV5-miHTT occurs, which mediates the widespread and sustained target engagement observed in our preclinical studies: (i) Initially, local administration results in significant miHTT expression in the surgically targeted brain regions, which are most affected by HD (putamen and caudate). (ii) The AAV is then transported to distant brain regions (e.g., the cortex) via axonal anterograde/retrograde transport. (iii) Because of its miR-451 scaffold, miHTT is actively and preferentially loaded into EVs, resulting in intercellular transport of miHTT, independently of neuronal connections, to adjacent cells.

To assess this hypothesis, we used cell culture systems to evaluate the functional transfer of miHTT to distant cells. We confirmed the transfer of miHTT derived from an overexpressing miHTT cell line or from AAV5-miHTT transduced HD patient iPSC-derived neurons to recipient cells takes place in a contactless system. Moreover, the increased levels of miHTT in recipient cells results in a significant reduction in *HTT* mRNA levels, indicating that miHTT is still active after transfer. miHTT is likely to be trafficked to recipient cells by EVs, given the colocalization of miHTT and EV markers (in immunocytochemical and in situ hybridization experiments) and that miHTT was present in EV-enriched fractions isolated from culture media. 

Taken together, these results suggest that EV-mediated transport contributes to the widespread brain biodistribution of miHTT following a one-time localized injection. This confirms our previous studies showing that EV-associated miHTT is secreted from AAV5-miHTT transduced HD neurons [47]. miHTT could also be detected in the CSF of NHPs up to two years post AAV5-miHTT administration in the caudate and putamen [47].

Supporting our hypothesis, endogenous miRNAs have been documented in all body matrices, including plasma, CSF, [51,52], and cell-conditioned media [47]. Numerous investigations have confirmed that the transfer of extracellular miRNAs to recipient cells can modulate gene expression [53]. Didiot et al. also demonstrated the ability of exosomes to widely disseminate engineered HTT-siRNA beyond the initial administration site. This preclinical study conducted in mice demonstrated that exosomes loaded with siRNA (but not siRNA alone) were distributed bilaterally—following unilateral striatal administration—in both the striatal and cortical regions, resulting in reduced HTT expression [54]. 

The ability of EVs to disseminate their therapeutic cargos to remote cells and tissues is also being evaluated in several clinical trials for other diseases, including melanoma, colorectal and lung cancer, and chronic kidney disease [55,56,57,58,59].

One advantageous property of the miR-451 scaffold is its enrichment in EVs; thus, the loading of miHTT into EVs is enhanced [30]. Furthermore, circulating EVs containing miR-451 are internalized by recipient cells in vivo [60]. Previous studies have shown that integrating an engineered siRNA into a pre-miR-451 scaffold can mediate selective packaging into EVs and that EV-siRNA mediated knockdown of targets with 10-fold less siRNA than lipid nanoparticles, implying that secreted EVs are highly effective vehicles for delivering RNA targeting moieties such as miRNAs [61].

The selection of an miR-451 scaffold as a platform for the AAV-mediated expression of miHTT confers several advantages in addition to its preferential loading into EVs. (i) miR-451 does not produce a passenger strand, which reduces off-target activity and may reduce toxicity [24,29,62]. Mature miHTT processing is known to harness a Dicer-independent pathway [24,29]. Studies conducted in HD patient-derived neurons confirm mature functional miHTT production was not associated with any passenger strand activity [29]. (ii) The use of miR-451 also reduces engagement of the endogenous miRNA biogenesis pathway. Endogenous gene expression can be dysregulated by engineered miRNAs [63]. This is thought to be caused by the depletion of intracellular factors required for the processing of pri- and pre-miRNAs. Dicer (a pivotal component of cellular RNAi processing) is not engaged when an miR-451 scaffold is used [64]. We have shown that in AAV5-miHTT transduced HD patient-derived neurons, mature miHTT expression accounted for <2% of all intracellular miRNAs, and no biologically relevant perturbation of endogenous miRNAs was observed [29]. (iii) Extensive complementarity of target RNA to miRNA is thought to increase the likelihood of target-directed miRNA degradation (TDMD) [65,66]. Unlike canonically produced miRNAs, miR-451 requires the respective slicer and trimmer activities of Ago2 and PARN to generate pre-miR-451 species, which are further 3′ end trimmed, producing mature isomiRs of different lengths [67,68,69]. A varied isomiR profile may, therefore, reduce TDMD-mediated miHTT destruction. 

Interestingly, the percentage of shorter miHTT isomiRs was higher in EV-enriched fractions (isolated from donor miHTT overexpressing cell conditioned media) than in donor or recipient (HEK-293T or HeLa) cells, although it did not reach statistical significance. It is possible that the miHTT is cleaved or processed within EVs; however, this would need additional testing to verify, and the mechanism(s) by which this might occur remains unknown.

Varied isomiR repertoires have also been documented by others [70,71]. Analysis of canonical miRNAs derived from the plasma exosomes of 46 donors revealed similar isomiR profiles, suggesting that isomiR production and their sorting into EVs are regulated [70]. We and others speculate that isomiRs may act in concert to regulate their mRNA target [71]. 

Overall, the use of an miR-451 scaffold reduces off-target activity and dysregulation of endogenous gene expression, and its efficient loading into EVs may increase the potency of AAV5-miHTT.

We demonstrated the transfer of miHTT from engineered donor cells to naïve recipient cells and the subsequent suppression of *HTT* mRNA levels. Control experiments confirmed that the miHTT transfer is not mediated by donor cell migration through the transwell membrane or AAV5-miHTT vector transduction of recipient cells. We infer that EVs (based on the colocalization of miHTT signals and EV surface markers in staining experiments) shuttle miHTT to recipient cells. However, we cannot exclude the possibility that miHTT may also be transferred by other means. We have previously shown that AAV5-delivered therapeutic miRNAs are released from neuronal cells in vitro in association with both EVs and protein complexes [47]. miRNAs are both stable and resistant to degradation [72], so the passive transfer of unencapsulated or miHTT/protein complexes may occur both in vitro and in vivo. Repeating these studies in the presence and absence of compounds that inhibit EV release, such as inhibitors of membrane-neutral sphingomyelinase, would further define the role of EVs in this process. Colocalization studies were conducted in an engineered cell line that overexpresses miHTT; thus, the loading of miHTT into EVs is demonstrated. Extending these experiments to staining of the recipient cell lines and patient-derived neuronal cells would help to confirm that miHTT is taken up by recipient cells within EVs. EVs obtained from the conditioned media of AAV5-miHTT transduced patient-derived neuronal cultures were added to naïve neuronal cells, resulting in the dose-dependent transfer of miHTT. However, it may be that even the lowest concentration of EVs used in our experiments represented a non-physiological dose that may not reflect the situation in vivo. 

## 5. Conclusions 

This proof-of-concept study confirmed that miHTT can be loaded into EVs and delivered (without cellular contact) to naïve recipient cells. Increased miHTT levels and significant reductions in *HTT* mRNA were observed in recipient cells, indicating that EV-trafficked miHTT maintains its therapeutic properties. Staining experiments co-localized signals for both EV surface markers and miHTT, confirming that EVs are in part responsible for miHTT transfer. We, therefore, conclude that the intercellular transfer of miHTT by EVs is one of the mechanisms that augments the sustained therapeutic spread of AAV5-miHTT following one-time administration.

## 6. Patents

Method and means to deliver miRNA to target cells. Publication Number: WO/2020/104469 Publication Date: 28 May 2020. International Application No.PCT/EP2019/081822. Sogorb-González, M. and Vallès A. are listed as inventors of this patent.

## Figures and Tables

**Table 1:** Antibodies used for immunocytochemistry.

Primary Antibodies	Description	Reference Number	Dilution	Secondary Antibodies	Reference Number	Dilution
**Anti-CD9**	Rabbit monoclonal	ab92726	1:500	Alexa Fluor^®^ 647	A-31573	1:750
**Anti-CD63**	Rabbit monoclonal	ab252919	1:500	Alexa Fluor^®^ 647	A-31573	1:750
**Anti-CD81**	Mouse monoclonal	ab70559	1:500	Alexa Fluor^®^ 488	R37114	1:750

## Data Availability

The data presented in this study are available in the article or Supplementary Material.

## Supplementary Materials

The following supporting information can be downloaded at: https://www.mdpi.com/article/10.3390/cells11172748/s1, Supplementary Materials, and Methods: Sequence information; Supplementary Figure S1: Cell migration exclusion experiment; Supplementary Figure S2: Immunocytochemistry (ICC) for the visualization of EVs; Supplementary Figure S3: Fluorescent in situ hybridization (FISH) staining for the visualization of miHTT miRNAs; Supplementary Figure S4: Negative and positive controls for ICC and FISH staining. Supplementary Table S1: miHTT-overexpressing stable cell line generation: qPCR assay CT values.
