# Supplementary material for: Functional Intercellular Transmission of miHTT via Extracellular Vesicles: An In Vitro Proof-of-Mechanism Study
Source: Cells. 2022 Sep 3;11(17):2748. doi: 10.3390/cells11172748 (PMC9455173; doi:10.3390/cells11172748)
Supplement: Supplementary file 1 [file cells-11-02748-s001.zip › cells-1819525-supplementary.pdf]

Supplemental Materials and Methods: Sequence information.

Sequence used for generating the stable cell line overexpressing miHTT:

TCTAGATATCGGCGCTATGCTTCCTGTGCCCCCAGTGGGGCCCTGGCTGGGATTTCATCATATACTGTAAGTTTGCGATGAGACACTACAGTATAGATGATGTA
CTAGTCCGGGCACCCCCAGCTCTGGAGCCTGACAAGGAGGACAGGAGAGATGCTGCAAGCCCAAGAAGCTCTCTGCTCAGCCTGTCACAACCTACTGACTG
CCAGGGCACTTGGGAATGGCAAGGAAGGACTTGAGGGACTCGAAGACGAGTCCCTCAAGTCCTCTCTTGCTATACCCAGAAAACGTGCCAGGAAGAGAACTC
AGGACCCTGAAGCAGACTACTGGAAGGGAGACTCCAGCTCAAACAAGGCAGGGGTGGGGGCGTGGGATTGGGGGTAGGGGAGGGAATAGATACATTTTCTC
TTTCCTGTTGTAAAGAAATAAAGATAAGCCAGGCACAGTGGCTCACGCCTGTAATCCCACCACTTTCAGAGGCCAAGGCGCTGGATCCAGATCTCGAGCGGCC
GCCCGTGGCATCCCTGTGACCCCTCCCCAGTGCCTCTCCTGGCCCTGGAAGTTGCCACTCCAGTGCCCACCAGCCTTGTCCTAATAAAATTAAGTTGCATCA

Sequences used for generating the (custom-made) fluorescent in situ hybridization (FISH) probes:

- 24nt: 5′CGTCTTCGAGTCCCTCAAGTCC3′
- 30nt: 5′CTCGTCTTCGAGTCCCTCAAGT3′

	Probes	
**Samples**	**miHTT**	**U6**
Wild-type HEK-293	*N/D	15.88
HEK293-miHTT	19.53	15.71
No template	*N/D	*N/D

Supplemental Table S1: miHTT-overexpressing stable cell line generation: qPCR assay and CT values.

A qPCR assay was performed using miHTT (assay ID #CTXGPY4) and endogenous U6 [a positive internal control gene for miRNA qPCR assays (assay ID #001973)] probes against the listed samples and negative controls; average Ct values are listed in **Supplemental Table S1**. None of the minus RT samples generated signal in qPCR assays. The Ct values obtained confirmed that the expression of miHTT could be detected in Flp-In™293-miHTT stable pool cells. *N/D indicates a non-detectable signal. qPCR for the listed samples was performed in triplicate.

(A)

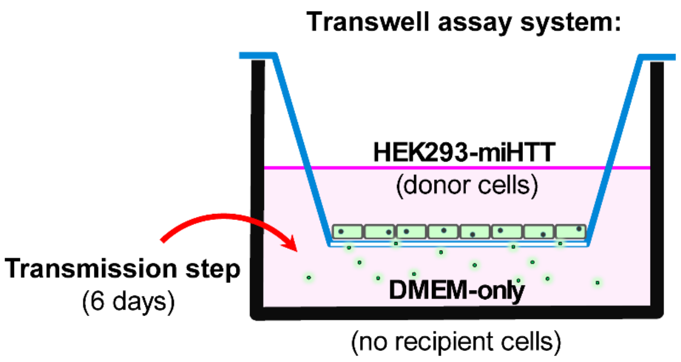

(B)

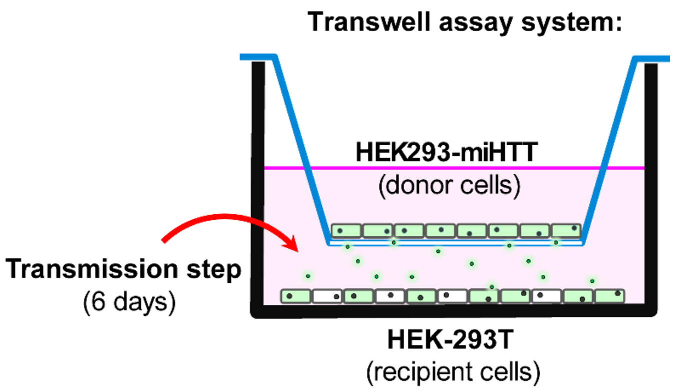

(C)

Sample Name	Nucleic Acid (ng/µL)	A260/A280	A260/A230	A260	A280	Nucleic Acid Factor	Baseline Correction (nm)	Baseline Absorbance
Negative control	-4.52	1.594	1.904	-0.113	-0.071	40	340	0.028
Negative control	-5.91	1.269	0.761	-0.148	-0.116	40	340	-0.04
Negative control	-4.21	1.657	2.761	-0.105	-0.064	40	340	0.013
Positive control	202.04	1.986	2.129	5.051	2.543	40	340	0.042
Positive control	345.56	1.979	2.001	8.639	4.365	40	340	0.136
Positive control	788.58	2.014	2.157	19.715	9.791	40	340	0.358

Supplemental Figure S1: Cell migration exclusion experiment.

A) Negative control samples; inserts containing the donor HEK293-miHTT cells were placed on top of wells containing only medium and maintained in culture for 6 days. **B)** Positive control samples; donor HEK293-miHTT cells were placed on top of wells containing HEK-293 wild-type cells in the bottom of the transwell plate and similarly maintained in culture for 6 days. **C)** Following RNA isolation, no nucleic acid was quantified by spectrophotometry in the medium-only negative control samples. This confirmed the absence of cell migration through the transwell insert.

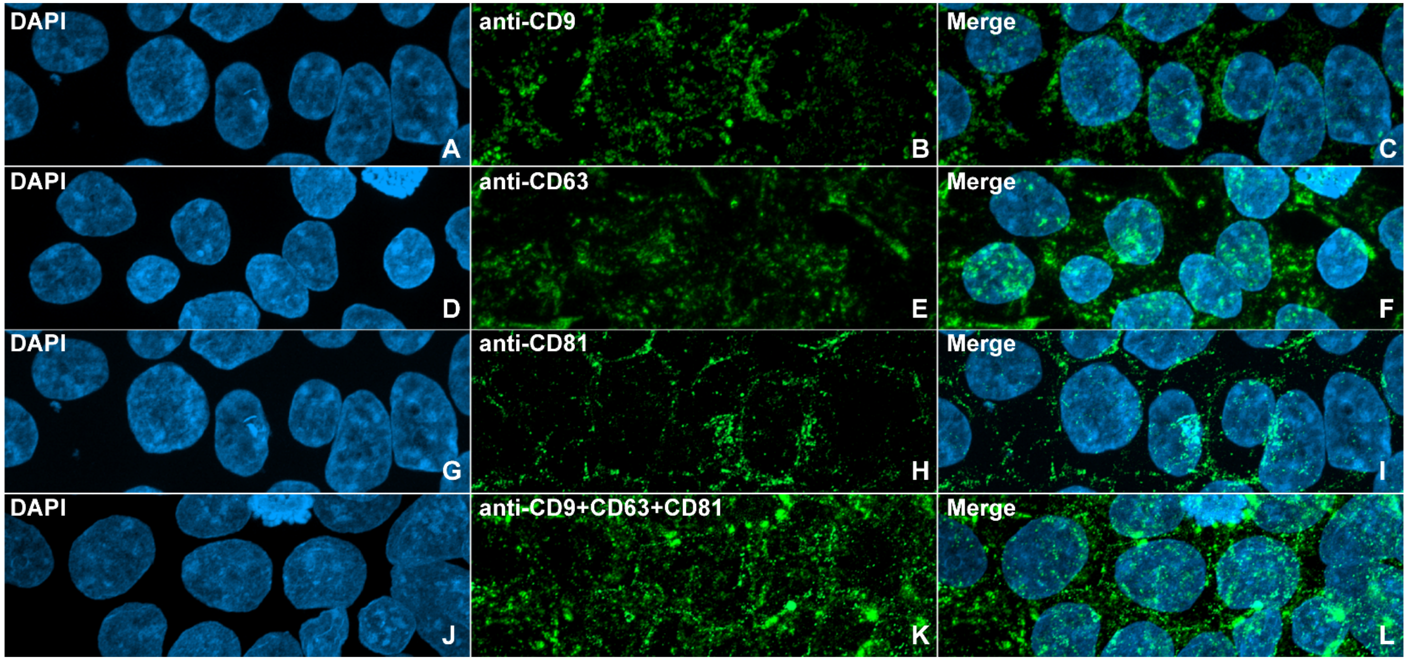

Supplemental Figure S2: Immunocytochemistry (ICC) for the visualization of EVs. Immunofluorescent staining of HEK293-miHTT cells using anti-CD9 (**A-C**), anti-CD63 (**D-F**), anti-CD81 (**G-I**), and a cocktail of antibodies (anti-CD9+CD63+CD81) (**J-L**). Alexa Fluor® 488 and/or 647 were used. For analysis, the color channel was switched to green when Alexa Fluor® 647 was used. Cells were counterstained with DAPI.

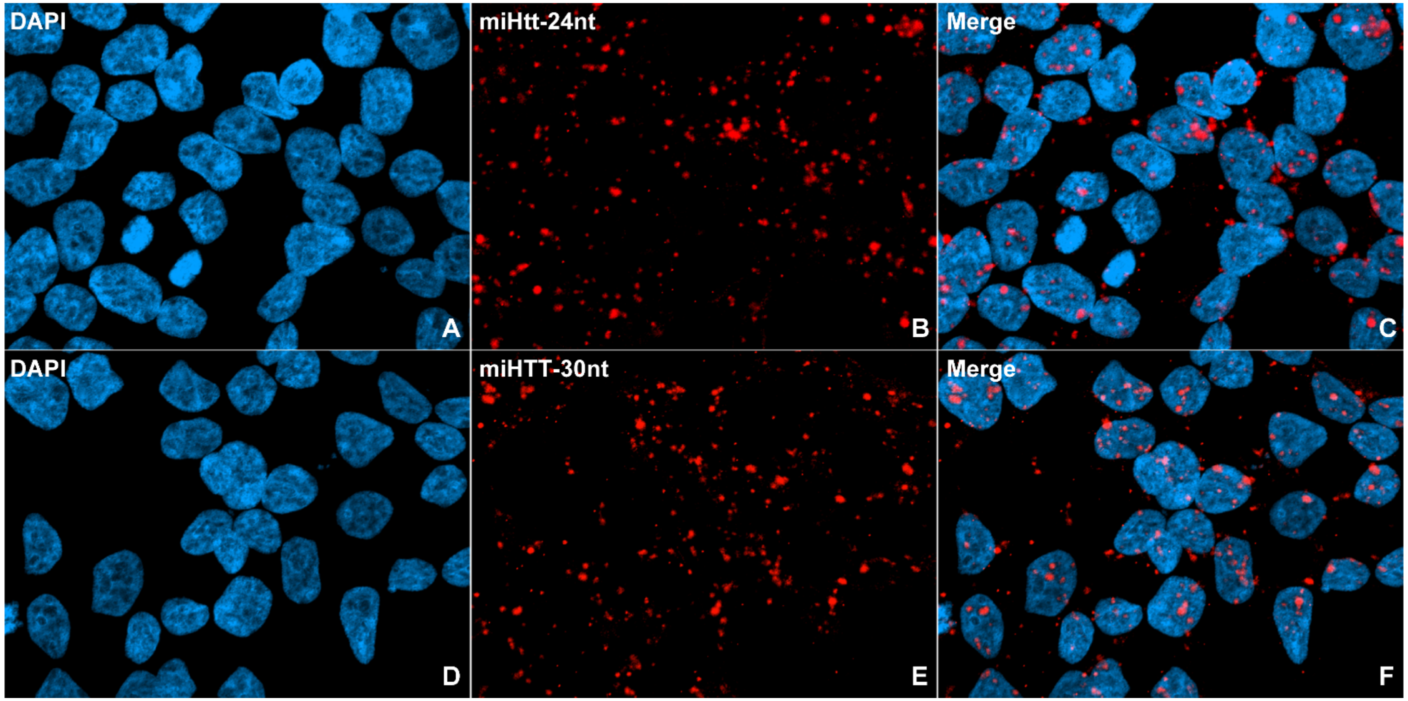

Supplemental Figure S3: Fluorescent in situ hybridization (FISH) staining for the visualization of miHTT. A-C) miHTT detection using the miHTT-24nt probe. **D-F**) miHTT detection using the miHTT-30nt probe. nt, nucleotide.

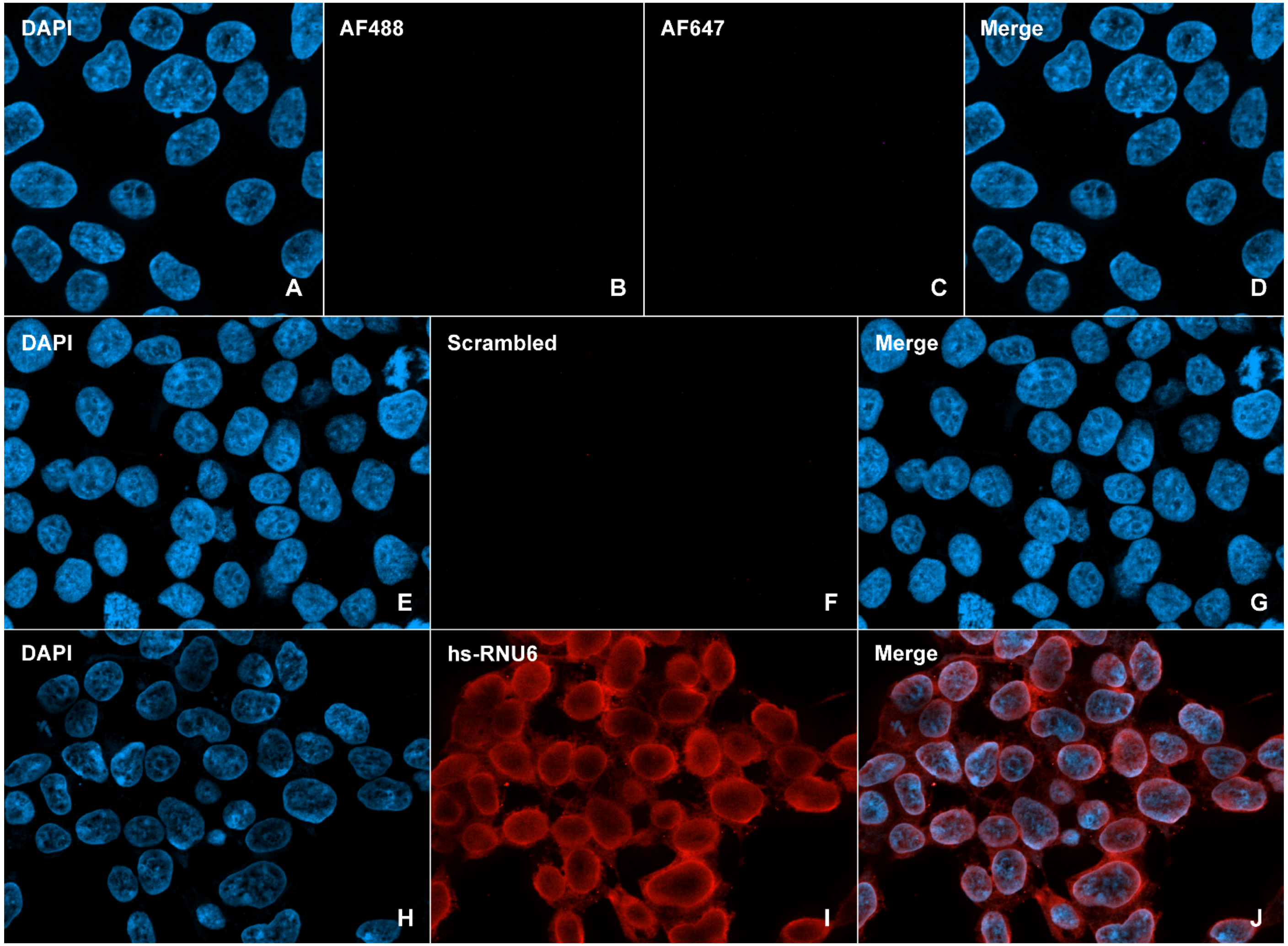

Supplemental Figure S4: Negative and positive controls for ICC and FISH staining. **A-D**) Treatment with the primary antibody was omitted as a negative control for ICC. **E-G**) Negative control for FISH staining was conducted with a scrambled probe. **H-J**) Positive control for FISH staining using the hs-RNU6 probe.
